# Cancer Pain Is Not One-Size-Fits-All: Evolving from Tradition to Precision

**DOI:** 10.3390/clinpract15100173

**Published:** 2025-09-23

**Authors:** Nidha Shapoo, Abdul Rehman, Carlos Izaguirre-Rojas, Vladimir Gotlieb, Noella Boma

**Affiliations:** Department of Medicine, New York Medical College/Metropolitan Hospital, New York, NY 10029, USA

**Keywords:** cancer pain, evolution, WHO analgesic ladder, emerging advances, biomarkers, precision medicine, personalized approach, challenges

## Abstract

Cancer pain remains a significant challenge in oncology, profoundly affecting patients’ quality of life, function, and prognosis. Historically under-recognized and managed with a uniform, opioid-centric approach, cancer pain was often inadequately treated. Advances in pain assessment, multimodal analgesia, and supportive care have improved outcomes, yet traditional algorithms frequently fail to address the complex, heterogeneous nature of cancer pain. Contemporary management is shifting toward precision and personalized medicine, integrating genetic, molecular, and biomarker data with individual patient characteristics to inform treatment decisions. To address this complexity, we propose a five-domain framework encompassing biological, pharmacologic, psychological, sociocultural, and functional domains. This multidimensional approach enables clinicians to tailor pain management strategies to each patient’s unique profile, aiming for equitable and individualized care. However, challenges remain, including tumor and patient heterogeneity, limited biomarker validation, data integration, disparities in access, and the need for multidisciplinary coordination. This review traces the evolution of cancer pain management, highlights the promise of precision and personalized strategies, and presents a comprehensive framework for optimizing pain control in oncology.

## 1. Introduction

Cancer pain is a complex and multifaceted symptom affecting about 40–70% of patients with cancer [1,2]. A recent meta-analysis by Snijders et al. showed a decline in the prevalence and severity of pain over the past decade. However, the prevalence remains high (54.6%), especially in advanced, metastatic, and terminal cancer patients [1]. Pain continues to be the most prevalent symptom during and after the cancer treatment and may be debilitating up to three months after curative treatment. However, patients who are not eligible for anti-cancer treatments frequently report moderate to severe pain [3]. Cancer pain significantly affects the patient’s quality of life, leading to emotional distress, cognitive dysfunction, and reduced survival rates [1,3,4,5].

A cohort study by Perez et al. estimated the prevalence of pain in long-term cancer survivors and found that at least 40% of cancer survivors had persistent pain. Pain was neuropathic in at least half of cancer survivors and a frequent health determinant, causing sleep disorders, mood alteration, and fatigue [6].

*“We can ignore even pleasure. But pain insists upon being attended to.”*

—C.S. Lewis

Managing cancer pain is critical to improving the quality of life and survival rates. Cancer pain management has evolved significantly over the decades due to increased awareness and knowledge, improved pain assessment tools, a multidisciplinary approach to cancer, and novel treatment strategies [1].

This review provides an in-depth analysis of the historical evolution of cancer pain, its pathophysiology and types, contemporary assessment techniques, conventional and advanced treatment modalities, and discusses how a personalized treatment approach can improve outcomes and patient experiences.

## 2. The Evolution of Cancer Pain: From Tradition to Personalization

Historically, cancer pain was overlooked. During the Middle Ages, it was considered a punishment that was managed through spiritual rituals, acupuncture, or herbal remedies.

The 19th century represented a pivotal moment with the isolation of morphine in 1806. Morphine remains the most widely used analgesic globally and is included in the World Health Organization’s List of Essential Medications [7]. In 1883, heroin was first available over the counter for pain management [8]. Opioids rapidly emerged as the preferred substances for pain management, sleep induction, mood enhancement, and the treatment of mental health disorders. This resulted in their extensive utilization, the emergence of adverse effects, morbidity, potentially fatal respiratory depression, and opioid dependence.

In 1914, the United States government acknowledged the risk of opioid abuse and enacted the Harrison Narcotics Act, which forbade the nonmedical use of prescribed opioids [9].

The opioid epidemic commenced in the mid-to-late 1990s. The phenomenon was marked by three phases: overdose fatalities from prescription opioids commenced an upward trajectory in 1996, attributed to heightened prescription rates and the launch of OxyContin. Heroin precipitated a second wave in 2010, particularly acute in the Northeast and South census regions. The rise in synthetic opioids, chiefly illicit fentanyl, initiated a significant third wave beginning in 2013. By the time the issue was designated a public health emergency on 26 October 2017, opioid overdose had already resulted in the deaths of hundreds of thousands of individuals [10].

The management of cancer pain has been complicated by the opioid crisis, resulting in increasing uncertainty regarding the provision of adequate pain relief while mitigating the risks of opioid misuse among cancer survivors. This has necessitated a thorough evaluation of both pain and potential substance use disorder risk factors by clinicians [11].

The World Health Organization’s Analgesic Ladder, established in 1986, serves as a fundamental framework for managing cancer pain, advocating a systematic method of pain reduction according to intensity using non-opioids, weak opioids, and potent opioids, thereby enhancing palliative cancer care [12]. Further innovations have occurred in the late 20th and 21st centuries, including patient-controlled analgesia, nerve blocks, neuromodulation, targeted therapies, integrative approaches, and an updated WHO ladder incorporating a fourth step. The field of palliative care has expanded to include a more holistic approach, focusing on the physical, emotional, and psychological aspects of cancer pain, making it a personalized experience for every patient [13,14].

## 3. Pathophysiology and Types of Cancer Pain

**Figure 1:**
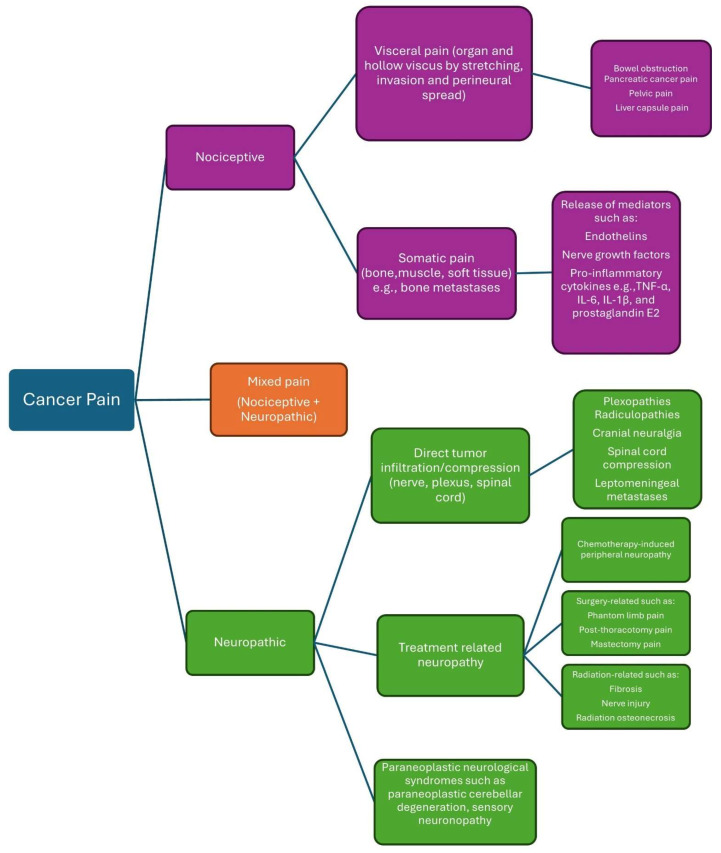
Pathophysiology of Cancer Pain.

Cancer pain is multifactorial, involving nociceptive (somatic or visceral) pain, neuropathic pain, and/or a mix of both (Figure 1) [15,16,17].

### 3.1. Nociceptive Pain

Nociceptive pain originates from the stimulation of nociceptors resulting from tissue injury, frequently induced by tumor infiltration into bones, muscles, or visceral organs.

Bone metastases represent the predominant etiology of cancer-related somatic pain and correlate with unfavorable prognosis and survival outcomes [18]. Approximately 75% of those with advanced cancer endure bone pain. Cancer-induced bone pain (CIBP) is a multifaceted pain condition characterized by inflammatory, neuropathic, ischemia, and cancer-specific causes [19]. Bone metastasis disturbs skeletal homeostasis by disrupting the equilibrium between osteoblastic bone production and osteoclast-mediated bone resorption [20]. Bone pain has been associated with osteoclast-mediated bone resorption. Tumor cells secrete endothelin, which promotes the proliferation of osteoblasts. Activated osteoblasts secrete receptor activators of nuclear factor-kappa-B ligand (RANKL), which stimulate the proliferation and maturation of osteoclasts, hence augmenting osteoclast-mediated degradation of bone matrix. Osteoclasts produce adenosine triphosphate and induce acidosis through proton release, leading to the activation of multiple receptors and ligand-gated ion channels, including P2X receptors, transient receptor potential V1 receptors, and acid-sensing ion channels type 3 found on bone-innervating sensory neurons. Tumor cells, stromal cells, and activated immune cells secrete several mediators, including endothelin, nerve growth factors, protons, and pro-inflammatory cytokines such as tumor necrosis factor TNF-α, IL-6, IL-1β, and prostaglandin E2, which activate nociceptors [21]. The acidic microenvironment is considered a crucial mechanism contributing to bone pain, as bone is a hypoxic tissue, resulting in increased cancer-induced acidity [22,23].

Nociceptors in the circulatory, pulmonary, gastrointestinal, and genitourinary systems, together with the head and neck, facilitate visceral pain perception. Tumors may induce irritation of mucosal and serosal surfaces, mesenteric torsion, and distension or contraction of a hollow viscus, which can activate nociceptors or lead to perineural invasion. Visceral pain is frequently widespread and inadequately localized. Visceral pain may be referred to as somatic pain due to the convergence of visceral and somatic signals within the central nervous system. Visceral pain may also activate autonomic nerve impulses and can be linked to nausea, sweating, or vasoconstriction [24,25]. Pain ranks as the third most common symptom among patients with pancreatic cancer, primarily resulting from perineural invasion [17]. Moreover, numerous genes associated with pain are increased in the dorsal horn of the spinal cord in murine models of pancreatic cancer, including Ccl12, Pin1, and Notum [26]. Mechanical stimulation in head and neck malignancies induces the persistent synthesis of serine proteases in the tumor microenvironment, resulting in pain [27].

### 3.2. Neuropathic Pain

Neuropathic pain affects 20–40% of cancer patients, manifesting as exclusively neuropathic in 20% and as a combination of neuropathic and nociceptive pain in 40%. Approximately two-thirds of neuropathic pain in cancer patients is attributable to direct tumor involvement; roughly 20% arises from cancer treatment, and 10–15% is due to comorbid conditions [28]. Neuropathic pain is characterized by a burning sensation; however, it may also present as diminished sensation or muscular weakness [29]. Neuropathic pain is more debilitating than nociceptive pain, necessitates higher doses of analgesics, and correlates with diminished physical and social functioning [6,30].

Neuropathic cancer pain due to direct tumor infiltration can occur due to nerve compression and ischemia, demyelination, and axonal degeneration, and include radiculopathies, plexopathies, cranial neuralgia, peripheral neuropathies, cancer-induced bone pain, leptomeningeal metastases, and spinal cord compression [29]. Cancer-induced immune responses can result in paraneoplastic neurological disorders, including paraneoplastic cerebellar degeneration and sensory neuronopathy [30].

Neuropathic pain generated by cancer treatment may arise from chemotherapy, surgical procedures, or radiation therapy. Chemotherapy-induced peripheral neuropathy (CIPN) is a prevalent, dose-limiting adverse effect of chemotherapy that may begin within the initial two months of treatment and persist for months or years following the cessation of chemotherapy. Chemotherapeutic medicines associated with peripheral neuropathy comprise platinum-based medications (cisplatin and oxaliplatin), taxanes (paclitaxel and docetaxel), vinca alkaloids (vincristine), thalidomide, and bortezomib. The mechanisms of CIPN encompass mitochondrial malfunction in sensory neurons, disruption of axonal transport, activation of sodium and calcium channels resulting in hyperexcitability, neuronal damage and inflammation, oxidative stress, and the production of inflammatory cytokines [29,30]. Surgical treatments may cause direct damage to peripheral nerves. In contrast, the mechanisms underlying radiation-induced neuropathy are not entirely understood. Nonetheless, they may arise from nerve compression resulting from radiation-induced fibrosis or direct nerve and blood vessel injury due to microvascular alterations [31].

### 3.3. Cancer Pain Syndromes

**Figure 2:**
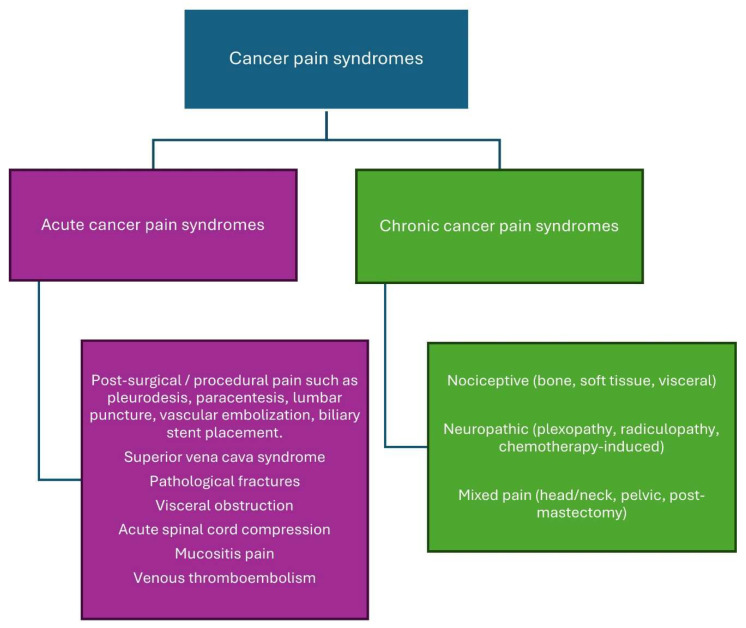
Cancer Pain Syndromes.

Cancer pain syndromes are categorized as acute or chronic depending on the onset and duration of pain (Figure 2). Understanding cancer pain syndromes is essential to know the etiology and prognosis of the pain and guide therapeutic interventions [32].

#### 3.3.1. Acute Cancer Pain Syndrome

Acute cancer pain syndromes are mainly associated with diagnostic or therapeutic interventions; however, they may also be caused by direct tumor infiltration or antineoplastic therapy. The diagnostic and therapeutic interventions that lead to acute pain include blood sampling, lumbar puncture, biopsy, injections, pleurodesis, chest tube insertions, paracentesis, percutaneous biliary stent placement, vascular embolization, suprapubic catheterization, and nephrostomy tube insertion. Acute pain syndrome caused by the tumor itself merits urgent intervention as it may result from bone metastases, pathological fracture, hemorrhage into the tumor, superior vena cava syndrome, venous thromboembolism, or obstruction/perforation of a hollow viscus. Antineoplastic therapy can cause acute pain syndrome due to mucositis, myalgias, arthralgias, bone pain, hand-foot syndrome, headaches or chemotherapy-induced peripheral neuropathy [32].

#### 3.3.2. Chronic Cancer Pain Syndrome

Chronic cancer-related pain is characterized as persistent pain resulting from the underlying malignancy or its metastases. Antineoplastic therapy may induce persistent pain resulting from neuropathy, arthralgias, osteoporosis, headaches, lymphedema, or post-surgical discomfort [32,33]. Chronic cancer pain has recently been categorized in the International Classification of Diseases (ICD-11) by the International Association for the Study of Pain (IASP) to enhance the formulation of personalized treatment strategies for affected patients and to promote research into these pain syndromes [34].

## 4. Assessment of Cancer Pain

Accurate assessment of cancer pain is essential for effective management. Cancer pain must be assessed at each clinical appointment, including a thorough pain history, physical examination, psychosocial evaluation, and relevant diagnostic tests. Self-reporting is the benchmark for evaluation; however, in practice, patients may hesitate to accurately disclose their pain due to apprehensions regarding side effects or addiction to analgesics, a desire to avoid ‘complaining’ about pain, efforts to ensure that physicians prioritize cancer treatment over symptom management, or misconceptions regarding the inevitability of pain [35,36].

### 4.1. Pain Assessment Tools

Patient-Reported Outcomes (PROs) in cancer pain are critical tools for understanding the patient’s subjective experience of pain. PROs guide treatment adjustments in real-time, improving quality of life.

Several validated PRO scales and tools can be used to assess cancer pain intensity:

#### 4.1.1. Unidimensional Pain Scales

Unidimensional scales are a straightforward method for assessing only one aspect of pain, specifically its intensity. These scales may help determine the severity of acute pain.

The Visual Analog Scale (VAS) and Numerical Rating Scale (NRS) are the predominant unidimensional pain assessment tools [37]. 

**Figure 3:**
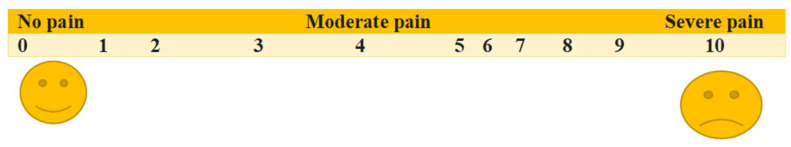
Visual Analog Scale.

The visual analog scale (VAS) is one of the most utilized pain assessment tools in the United States. It consists of a horizontal (or vertical) line, usually 10 cm in length, the left end of which signifies no pain, while the right end signifies the worst possible pain. This visual depiction of pain levels helps patients communicate the intensity of their pain. The distance is measured in millimeters and interpreted as follows: no pain, 1 to 3 cm; mild pain, 4 to 6 cm; moderate pain, 7 to 10 cm; and severe pain (Figure 3).

In the NRS, patients may be instructed to circle numerals evenly distributed on a sheet or to vocally assess pain intensity utilizing a scale from 0 to 10, where 0 signifies “no pain” and 10 denotes “the most excruciating pain conceivable.”

Numeric scales have advantages such as simplicity, consistency, and sensitivity to little variations in pain; yet, they are insufficient for evaluating neuropathic pain, which necessitates specialized scales [38].

#### 4.1.2. Multidimensional Pain Scales

Multidimensional pain scales have been created to represent the complexity of the pain experience. They are more intricate; however, they assess the severity, character, and location of the pain, along with its influence on activities or mood. These are especially beneficial in situations involving complex or persistent acute or chronic pain. The instruments are the McGill Pain Questionnaire (MPQ) and the Brief Pain Inventory (BPI).

**McGill Pain Questionnaire (MPQ)**

The MPQ offers a quantitative assessment of pain, facilitating the differentiation between its sensory and emotional components. Detecting a reaction to treatment can also be advantageous. The assessment comprises four sections and requires 5 to 15 min for completion. It mainly comprises several adjectives employed to characterize pain across three dimensions: sensory, emotional, and evaluative. The words are categorized into 20 subclasses arranged to denote relative intensity. The distribution, temporal pattern, and degree of pain are also assessed [39].

**Brief Pain Inventory (BPI)**

The BPI measures both the degree of pain and its interference with functionality. It is utilized for patients with cancer, human immunodeficiency virus, and arthritis. The assessment requires 5–15 min to complete and employs 11 quantitative scales to evaluate pain severity, mood, work capacity, interpersonal connections, sleep quality, life satisfaction, and the influence of pain on overall activity. The Brief Pain Inventory assesses the progress of a patient with a progressive disease, revealing whether there is an enhancement or deterioration in their mood and activity levels. Assessing function is crucial in comprehensive pain management [40].

In patients with cognitive impairments, monitoring pain-related behaviors, including facial expressions, body movements, verbalizations, or vocalizations, together with alterations in interpersonal relationships, serves as an alternate method for evaluating the existence of pain (albeit not its intensity). Various observational scales exist in the literature; however, none have been validated in other languages [41,42].

## 5. Treatment of Cancer Pain

Accurate diagnosis of the cause and characteristics of pain is essential for appropriate pain management in cancer patients and survivors. The concept of metamorphic pain management, which integrates interdisciplinary care teams, helps tailor treatment, provide innovative options, and offer better supportive care [43].

WHO has developed guidelines (the last set issued in 1996) for the pharmacological and radiotherapeutic management of cancer pain, providing evidence-based guidance for initiating and managing cancer pain.

The clinical guidelines and recommendations are categorized into three primary areas:

- Management of cancer pain: This pertains to the selection of analgesic medications for initial pain relief and the selection of opioids for ongoing pain management, encompassing the optimization of rescue medications, routes of administration, and the processes of opioid rotation and discontinuation.
- Adjuvant pharmacotherapy: This encompasses the administration of steroids, antidepressants, and anticonvulsants as adjunctive medications [44].
- Management of pain associated with bone metastases: This includes the administration of bisphosphonates and radiotherapy for the treatment of bone metastases.

### 5.1. The Original WHO Analgesic Ladder

The Original WHO analgesic ladder, introduced in 1986, continues to be the foundation of cancer pain management: This entails a three-step methodology [Table 1] [12].

The WHO has also given recommendations on the proper use of analgesics for pain [Table 2]. 

### 5.2. The Revised WHO Analgesic Ladder

The updated WHO analgesic ladder incorporates a fourth step and considers neurosurgical interventions, including brain stimulators. Invasive treatments, including nerve blocks and neurolysis (e.g., phenolization, alcoholization, thermocoagulation, and radiofrequency), are employed at the fourth step [45,46,47]. The newly proposed fourth step is advised for the management of chronic pain.

This revised analgesic ladder incorporates more opioids, including tramadol, oxycodone, hydromorphone, and buprenorphine, as well as novel administration methods, such as transdermal patches, which were unavailable in 1986 [48]. Methadone, in step 3, is essential as it plays a pivotal role in managing cancer pain and is also highly effective in opioid rotation [47,49].

Adjuvant drugs for neuropathic pain encompass steroids, anxiolytics, antidepressants, hypnotics, anticonvulsants, gabapentinoids (gabapentin and pregabalin), membrane stabilizers, sodium channel blockers, and N-methyl-d-aspartate (NMDA) receptor antagonists. Cannabinoids can be classified among adjuvant drugs, since they serve as adjuncts in the treatment of palliative cancer patients and individuals with AIDS, while also enhancing the quality of life for those suffering from chronic pain. They may also be utilized for the management of chronic neuropathic pain [50,51].

This iteration of the analgesic ladder can be employed bidirectionally: the gradual ascent for chronic and cancer-related pain, and the rapid descent for severe acute pain, uncontrolled chronic pain, and breakthrough pain. This proposal’s advantage is in the ability to ascend gradually, one step at a time, in cases of chronic pain, and, if required, to accelerate the pace of progression based on pain intensity. In extreme circumstances, one may commence straight at the fourth stage to manage severe pain by utilizing patient-controlled analgesia pumps for continuous intravenous, epidural, or subdural delivery. Upon achieving pain control, one may transition to medications from step 3.

### 5.3. Pharmacological Options

#### 5.3.1. Non-Opioid Analgesics (Table 3)

##### Nonsteroidal Anti-Inflammatory Drugs

Nonsteroidal anti-inflammatory medications (NSAIDs) are analgesic and antipyretic agents that inhibit cyclooxygenase (COX-1/COX-2) to reduce prostaglandin synthesis; they comprise nonselective agents (ibuprofen, naproxen, diclofenac, ketorolac, indomethacin) and COX-2-selective drugs (celecoxib). Most are chemically weak organic acids characterized by significant protein binding, hepatic metabolism by CYP2C9 and glucuronidation, and renal excretion of metabolites. In cancer-related pain, particularly bone and inflammatory pain, they are opioid sparing. Typical administration routes include oral (all), intravenous/intramuscular (ketorolac; intravenous ibuprofen), rectal (diclofenac/indomethacin), and topical (diclofenac) if indicated. Toxicity is dose-dependent; ibuprofen doses of ≥100 mg/kg frequently induce symptoms, while doses of ≥400 mg/kg provide a risk of severe toxicity. Adverse effects of NSAIDS include dyspepsia, gastritis/ulcer/bleeding, acute kidney injury and sodium retention (edema, hypertension, heart failure exacerbation), cardiovascular thrombotic events (myocardial infarction/stroke, elevated risk with COX-2 selective agents and diclofenac), hepatotoxicity (particularly associated with diclofenac), bronchospasm in aspirin-exacerbated respiratory conditions, reversible platelet inhibition (absent with COX-2 agents), and delayed bone healing. Contraindications include active gastrointestinal bleeding or peptic ulcer, advanced chronic kidney disease (eGFR < 30 mL/min/1.73 m^2^), decompensated heart failure, recent coronary artery bypass grafting, third-trimester pregnancy, and hypersensitivity to NSAIDs or aspirin. NSAIDs have ceiling effects with no therapeutic gain and increased adverse effects by increasing doses beyond recommended dosages, unlike opioids that can be titrated for pain relief [52].

**Table 3:** Summary of Non-Opioid Medications for Cancer Pain.

Drug Class	Examples	Best for
NSAIDS	Ibuprofen 400–800 mg every 6–8 h (maximum 2.4–3.2 g/day Naproxen 250–500 mg every 12 h (maximum 1–1.5 g/day) Diclofenac 50 mg three times daily (maximum 150 mg/day) Ketorolac 15–30 mg intravenously/intramuscularly every 6 h (maximum 120 mg/day; usage for no more than 5 days) Celecoxib 100–200 mg twice daily (maximum 400 mg/day).	Mild pain, Bone pain, and Inflammation.
Acetaminophen	Paracetamol 325–650 mg q4–6h or up to 1000 mg q6h (max 3–4 g/day)	Mild pain, adjunct use.
Antidepressants	Duloxetine 30–60 mg/day Amitriptyline 10–75 mg at bedtime Nortriptyline 25–75 mg/day	Neuropathic pain
Anticonvulsants	Gabapentin 100–300 mg three times daily starting dose, can go up to 3600 mg/day Pregabalin 75 mg twice daily starting dose, can go up to 600 mg/day	Neuropathic pain
Corticosteroids	Dexamethasone 2–4 mg daily for mild symptoms; up to 8–16 mg/day (divided into twice daily) Prednisone 10–60 mg/day orally Methylprednisolone 16–32 mg/day, sometimes in divided doses.	Bone pain, edema, nerve compression, raised intracranial pressure, malignant spinal cord compression.
NMDA receptor antagonists	Ketamine 0.1–0.5 mg/kg/h by infusion, 0.25–0.5 mg/kg every 8–12 h oral, or intravenous bolus 0.1–0.25 mg/kg. Amantadine 100 mg once or twice daily (max 400 mg/day)	Refractory neuropathic pain
Cannabinoids	THC, CBD 2.5–20 mg/day	Neuropathic pain, appetite stimulant, anxiolytic
Bisphosphonates	Zoledronic acid; 4 mg IV over at least 15 min every 3–4 weeks, adjusted for renal function Pamidronate; 90 mg IV over 2 h every 3–4 weeks	Bone metastases, Bone pain
RANKL inhibitors	Denosumab; 120 mg subcutaneously every 4 weeks	Bone metastases, Bone pain
Topical agents	Lidocaine patch, Capsaicin	Localized neuropathic pain
Antispasmodics and Muscle Relaxants	Baclofen, 5 mg orally three times daily, titrated up as tolerated (max 80 mg/day). Hyoscine; 20 mg orally or IV every 6–8 h as needed	Muscle-related pain, visceral pain.
Novel drugs	Haloperidol; 0.5–2 mg orally once or twice daily Mirogabalin; 10–15 mg orally twice daily (maximum 30 mg twice daily), adjusted for renal impairment. PEA; 300–600 mg orally two to three times daily. Clonidine; 0.1–0.3 mg twice daily oral, 0.1–0.3 mg/day release transdermal, 30–300 µg/day epidural infusion.	Neuropathic pain, oxaliplatin-induced neuropathy, adjunct use.

##### Acetaminophen (Paracetamol)

Acetaminophen (paracetamol) is an analgesic and antipyretic from the aniline derivative class, distinct from NSAIDs as it has little anti-inflammatory activity. It acts mainly via central COX inhibition, particularly COX 2, and serotonergic pathways. It is a weakly acidic compound, metabolized in the liver by glucuronidation/sulfation, with a minor CYP2E1 pathway forming N-acetyl-p-benzoquinone imine, the toxic metabolite detoxified by glutathione. The routes of administration include oral, rectal and intravenous. Acetaminophen is associated with hepatotoxicity at higher doses, so doses should not exceed 4000 mg per day. The adverse effects include hepatotoxicity, rare hypersensitivity, and renal injury at high doses. Acetaminophen is contraindicated in severe liver disease, chronic alcohol use and in cases of hypersensitivity. In cancer pain, it is often combined with opioids as a safe, opioid-sparing agent with minimal gastrointestinal and renal toxicity [53].

##### Adjuvant Analgesics

Antidepressants, including serotonin-norepinephrine reuptake inhibitors (SNRIs) such as Duloxetine, tricyclic antidepressants (TCAs) like Amitriptyline and Nortriptyline, and anticonvulsants such as Gabapentin and Pregabalin, are employed to manage neuropathic pain and serve as adjunct therapies alongside non-opioid and opioid medications for enhanced efficacy. Duloxetine (SNRI) is a centrally acting analgesic used orally in a dose of 30–60 mg/day. Overdose may cause serotonin syndrome, seizures and hepatotoxicity. Adverse effects include nausea, dizziness, insomnia, sweating and rise in blood pressure. Duloxetine is contraindicated in severe liver disease, alcohol abuse and narrow-angle glaucoma. Amitriptyline (TCA) is a tertiary-amine antidepressant used at a dose of 10–75 mg at bedtime. The adverse effects include sedation, dry mouth, constipation, urinary retention, weight gain and QT prolongation. Nortriptyline (TCA) is a secondary-amine and an active metabolite of amitriptyline. Nortriptyline is used at an oral dose of 25–75 mg/day. The side effect profile is like amitriptyline. Gabapentin is an anticonvulsant modulating calcium channels with adverse effects including dizziness, sedation, edema and weight gain. Gabapentin should be avoided with drugs causing central nervous system depression and in renal impairment.

Pregabalin is another anticonvulsant modulating calcium channels with adverse effects including dizziness, somnolence, edema, blurred vision, weight gain and euphoria. The dose of pregabalin needs to be modified in renal impairment [53,54]

##### Corticosteroids

Corticosteroids, including dexamethasone, prednisone, and methylprednisolone, are commonly used as adjuvant agents in cancer pain management, particularly for pain associated with bone metastases, spinal cord compression, nerve compression, raised intracranial pressure, hepatic capsular distension, and inflammation. They provide analgesic benefits by reducing peritumoral edema, suppressing inflammatory mediators, and alleviating pressure-related pain. Corticosteroids also cause neuroimmune modulation that may help neuropathic pain. However, their long-term use is limited by significant adverse effects such as hyperglycemia, immunosuppression, muscle weakness, mood changes, osteoporosis, and gastrointestinal bleeding, with heightened concern in elderly and frail patients [55].

##### N-Methyl-D-Aspartate (NMDA) Receptor Antagonists

Ketamine is an NMDA receptor antagonist used in low, sub-anesthetic doses for refractory neuropathic and opioid-resistant cancer pain. Ketamine is a phencyclidine derivative, highly lipophilic and metabolized hepatically to active nor ketamine. It can be given intravenously, subcutaneously, orally, or intranasally; typical palliative care doses are 0.1–0.5 mg/kg/h by infusion, oral 0.25–0.5 mg/kg every 8–12 h, or intravenous bolus 0.1–0.25 mg/kg. The adverse effects of ketamine include hallucinations, dysphoria, dizziness, hypertension, tachycardia, hepatotoxicity, and cystitis with chronic oral intake [53]. Amantadine is another NMDA receptor antagonist and antiviral agent that has shown modest benefit in neuropathic pain management, including in some cancer patients with opioid-refractory pain. Amantadine is used at a dose of 100 mg once or twice daily (max 400 mg/day), adjusted for renal function. Adverse effects include dizziness, insomnia, livedo reticularis, dry mouth, constipation, hallucinations, and peripheral edema [56].

##### Cannabinoids

Cannabinoids such as delta-9-tetrahydrocannabinol (THC) and cannabidiol (CBD) act on CB1 receptors in the CNS and CB2 receptors in immune cells to provide analgesia, appetite stimulation, and anxiolysis. They are given orally or by inhalation, with therapeutic doses typically 2.5–20 mg/day THC or CBD equivalents. Excessive doses may cause sedation, hallucinations, or cardiovascular effects. The adverse effects include dizziness, drowsiness, cognitive impairment, and psychiatric symptoms. Their role in cancer pain, especially neuropathic pain, is limited by side effects and legal restrictions [57].

##### Bisphosphonates and RANKL Inhibitors

Bisphosphonates are used in oncology due to their ability to reduce skeletal complications. They exhibit a strong affinity for hydroxyapatite in bone, diminish osteoclast activity, and impede bone resorption, therefore reducing bone pain and skeletal-related events (SREs). Besides that, bisphosphonates also exert direct antitumor and antiangiogenic effects, thereby delaying the development of bone metastases. The most commonly used bisphosphonates include zoledronic acid and pamidronate. These drugs are generally well tolerated, but adverse effects may include acute-phase reactions such as fever, myalgia, flu-like symptoms, renal toxicity, electrolyte disturbances (hypocalcemia, hypophosphatemia, hypomagnesemia), and osteonecrosis of the jaw (rare and serious). Regular monitoring of renal function and dental health, along with supplementation of calcium and vitamin D, is recommended during therapy [58,59].

Denosumab is a fully human monoclonal antibody against RANKL, a key regulator of osteoclast activity. By blocking the RANKL–RANK pathway, it inhibits osteoclast formation and bone resorption, reducing skeletal-related events and bone pain in patients with bone metastases. It is given as 120 mg subcutaneously every 4 weeks with calcium and vitamin D supplementation. Unlike bisphosphonates, denosumab does not require renal dose adjustment, making it suitable for patients with renal impairment. Adverse effects include hypocalcemia, hypophosphatemia, osteonecrosis of the jaw, atypical femoral fractures, and rebound vertebral fractures after discontinuation [60,61].

##### Topical Agents

Topical analgesics, like 8% capsaicin and 5% lidocaine patches, serve as effective alternatives for pain management and are integral to multimodal analgesia. They have the capacity to inhibit the pain pathway locally or peripherally, while minimizing systemic absorption. They are easy to use, providing direct access to target areas with little systemic side effects and are predominantly efficacious in neuropathic pain [62].

##### Antispasmodics and Muscle Relaxants

Antispasmodics and muscle relaxants, such as baclofen and hyoscine, are used as adjuncts for muscle-spasm–related and visceral pain. Baclofen is a GABA-B receptor agonist used at a starting dose of 5 mg orally three times daily, titrated up as tolerated (max 80 mg/day). The most common adverse effects with baclofen are sedation, dizziness, weakness, confusion, and hypotonia. Hyoscine, an anticholinergic agent, reduces smooth muscle spasm and colicky visceral pain. Side effects include dry mouth, blurred vision, constipation, urinary retention, and tachycardia [56].

##### Novel Non-Opioid Drugs

Haloperidol, a first generation anti-psychotic, is a high-affinity sigma-1 receptor antagonist, reducing central sensitization and neuroinflammation, thereby providing analgesic benefit in neuropathic pain, fibrosis-related pain, and radiation necrosis. Haloperidol is used at low doses as an adjunct to opioids with a side effect profile of sedation, extrapyramidal symptoms, QT prolongation, orthostatic hypotension, constipation, and tardive dyskinesia or neuroleptic malignant syndrome with long-term use [63].

Mirogabalin besylate is a gabapentinoid approved in Japan (2019) for the treatment of neuropathic pain. Like pregabalin, it binds to the α2δ subunit of presynaptic voltage-gated calcium channels, reducing calcium influx and inhibiting excitatory neurotransmitter release. It has shown benefit in cancer-related neuropathic pain, including pancreatic cancer and oxaliplatin-induced neuropathy. Common adverse effects include dizziness, headache, and constipation [63].

Palmitoylethanolamide (PEA) is an endogenous bioactive lipid of the ALIAmide (autacoid local injury antagonist amide) family that modulates neuroinflammation by downregulating mast cell activation and glial cell hyperactivity. Through these actions, it reduces non-neuronal inflammatory responses associated with neuropathic pain and systemic inflammation. Clinical studies suggest benefit in oxaliplatin-induced neuropathy and as an adjunct to opioids for pain control. The usual dose is 300–600 mg orally two to three times daily. PEA is generally well tolerated, with reported adverse effects being gastrointestinal upset, headache, or drowsiness [63].

Clonidine is an α2-adrenoceptor and imidazoline-2 receptor agonist that reduces sympathetic outflow and enhances descending inhibitory pain pathways, thereby attenuating neuropathic and cancer-related pain. It can be given orally (0.1–0.3 mg twice daily), trans dermally (weekly patch, 0.1–0.3 mg/day release), or via epidural infusion (30–300 µg/day) in refractory cases. Side effects include hypotension, bradycardia, sedation, dry mouth, dizziness, and constipation [63].

#### 5.3.2. Opioid Analgesics

Opioids are fundamental in the management of cancer-related pain due to their efficacy, ease of dosage adjustment, and dependability. Opioids function by binding opioid receptors (delta, kappa, or mu), predominantly the mu opioid receptors (MORs), located in both the peripheral and central neurological systems. Opioids constitute the primary treatment for moderate to severe cancer-related pain [64]. Opioids are efficacious analgesics for breakthrough cancer pain, characterized as a temporary exacerbation of pain occurring against a backdrop of adequately managed baseline pain, of moderate to severe intensity, with rapid onset (minutes), and relatively brief duration (median, 30 min) [65].

Despite the effectiveness of opioids, the opioid epidemic, due to misuse and addiction, created tension between effective pain relief and safety/regulatory concerns. Cancer patients faced challenges post-epidemic because of stricter prescribing laws, fear among physicians and pharmacists, and payer restrictions leading to under-prescription, premature tapering, insurance denials, and refusal to fill even appropriately prescribed opioids [10,11].

Opioid analgesics are categorized as either agonists, antagonists, or partial agonists depending on the response they produce after interacting with the opioid receptors.

The most used opioid analgesics for cancer pain include morphine, methadone, buprenorphine, fentanyl, hydromorphone, oxycodone, tramadol, codeine, and dihydrocodeine. Tramadol, codeine, and dihydrocodeine are weak opioids and are used for mild to moderate pain in combination with non-opioid analgesics. Strong opioids such as morphine, methadone, buprenorphine, fentanyl, hydromorphone, and oxycodone constitute the cornerstone of analgesic treatment for moderate to severe cancer pain. Tapentadol is a novel analgesic class that functions as a µ-opioid receptor agonist and noradrenaline reuptake inhibitor, seen as an alternative to morphine and oxycodone, particularly in cases of opioid toxicity [66]. Morphine, methadone, and fentanyl patches are classified as important medications in the WHO’s list of analgesics for cancer-related pain [67].

##### Tramadol

Tramadol is a centrally acting analgesic often used for mild to moderate cancer pain, classified as step 2 in the WHO analgesic ladder. Tramadol has a dual mechanism of action: it is a weak agonist of μ-opioid receptors and inhibits the reuptake of norepinephrine and serotonin [68]. Tramadol has also been found to be effective in neuropathic cancer pain and appears to improve the quality of life [69]. Tramadol may be used as a bridge between non-opioids and potent opioids for moderate pain and offers some benefits in terms of a lower side-effect profile, like less constipation, lower risk of respiratory depression, and safer use in elderly patients. The limitations of tramadol use include lowered seizure threshold, mainly when used with antidepressants, increased risk of serotonin syndrome, variable metabolism, and limited potency for moderate to severe cancer pain. Tramadol is used at a starting dose of 25–50 mg every 6 h with a maximum dose of 400 mg per day [68].

##### Codeine

Codeine is a weak opioid analgesic and, like tramadol, is used at step 2 of the WHO analgesic ladder. Codeine is a prodrug and is metabolized to morphine in the liver. Codeine is generally advised at dosages ranging from 30 mg to 60 mg for the management of mild to moderate pain. In most individuals, 5% to 10% of codeine is metabolized into morphine; a 30 mg dose of codeine is deemed like a 3 mg dose of morphine. Research demonstrates that codeine is superior to a placebo in alleviating cancer pain; nevertheless, it is associated with heightened risks of nausea, vomiting, and constipation, as well as insufficient analgesia for more intense cancer pain [70].

##### Dihydrocodeine

Dihydrocodeine is another weak opioid used for mild to moderate cancer pain. Dihydrocodeine primarily exerts its analgesic action through μ-opioid receptors, with lesser effects through κ-opioid receptors and δ-opioid receptors. Dihydrocodeine is more effective than tramadol for cancer pain and twice as potent as codeine. Its metabolite, dihydromorphine, is twice as potent as morphine [71].

##### Morphine

Morphine is predominantly a MOR agonist but also acts on delta and kappa receptors. Morphine is used at step 3 of the WHO analgesic ladder, may be used when there is an inadequate response to step 2 analgesics, for end-of-life/palliative care, bone metastases, visceral pain, and dyspnea in cancer. Oral morphine is the first choice for moderate to severe cancer pain because of its wide availability, cost-effectiveness, and decades of clinical use and evidence. Morphine possesses a brief half-life and is predominantly converted in the liver into two active metabolites: morphine-3-glucuronide (M3G) and morphine-6-glucuronide (M6G). Morphine is offered in several formulations, including oral tablets, oral liquid, suppositories, and solutions for intravenous (IV) and subcutaneous (SC) administration, rendering it a preferred opioid among clinicians. The constraints of morphine administration encompass tolerance and dependency, necessitate caution in cases of renal impairment, and are associated with adverse effects such as nausea, constipation, and sedation [72].

##### Oxycodone

Oxycodone is a MOR agonist with some kappa receptor activity used for moderate to severe cancer pain as an alternative to morphine. Oxycodone has higher oral bioavailability than morphine and can be safely used in renal dysfunction and in patients who are intolerant to morphine. The side effect profile of oxycodone is like morphine [73].

##### Hydromorphone

Hydromorphone is a pure MOR agonist and five to seven times more potent than morphine when administered orally. Like morphine, multiple dosage forms of hydromorphone are available, including oral liquid, oral tablet, suppository, and solution for IV or SC use. Hydromorphone is a preferred potent opioid for severe or escalating pain, safer in renal impairment, allows rapid titration, and is effective in small doses. Oral hydromorphone is 4–5 times stronger than oral morphine, whereas IV formulation is seven times stronger than IV morphine [74].

##### Fentanyl

Fentanyl is a selective MOR agonist used as maintenance for severe and chronic cancer pain. It is highly lipophilic and available in IV, transdermal, and oral transmucosal formulations. Transdermal fentanyl may be preferred over oral opioids in cases of poor gastrointestinal absorption, dysphagia, or constipation. Fentanyl is 80–100 times more potent than morphine and can be safely used in renal dysfunction. For opioid -tolerant patients, the transdermal patch starts at a dose of 12–25 mcg/hour and is changed every 72 h. The transdermal patch is extremely heat sensitive. External heat exposure increases the absorption of fentanyl, leading to potential overdose and respiratory depression. Transmucosal, buccal, and nasal forms are used for breakthrough cancer pain [75].

##### Methadone

Methadone is a long-acting opioid with MOR agonist and NMDA receptor antagonist properties. Methadone also inhibits the reuptake of serotonin and norepinephrine. These properties make methadone a suitable option for cancer patients with neuropathic pain in addition to nociceptive pain. Methadone is equally effective to morphine for cancer pain with higher oral bioavailability, lower cost, longer duration of action, and safer use in renal dysfunction. The toxicity profile of methadone is like that of morphine and fentanyl. Methadone causes QT prolongation in a dose-dependent manner, so ECG monitoring is required in patients who require > 30 mg/day [76]. The fact that methadone alleviates opioid cravings makes it a suitable option to use in opioid addiction treatment [77].

##### Tapentadol

Tapentadol is a new class of analgesics having a central mechanism of action with synergistic MOR agonist and noradrenaline reuptake inhibitory actions. It is efficacious for both opioid-naive patients and those currently utilizing opioids. Its reduced µ-opioid receptor binding affinity results in diminished opioid-related toxicities, including constipation and nausea. Tapentadol has demonstrated efficacy as an analgesic in many investigations. It should therefore be regarded as an alternative to morphine and oxycodone, particularly when opioid toxicities are a concern [66].

#### 5.3.3. American Society of Clinical Oncology (ASCO) Guidelines on Use of Opioids for Cancer Pain

The following clinical practice guideline recommendations were developed based on a systematic review of the medical literature [Table 4] [78].

#### 5.3.4. Choice of Opioid for Cancer Pain

Selecting the right opioid and dose is key to safe and effective cancer pain control. This requires careful assessment of patients to determine the type and severity of pain, history of opioid use, renal or hepatic dysfunction, cognitive status, and the route of administration required.

In opioid-naïve patients with moderate to severe pain, any short-acting selective MOR agonist may be used at low doses [Table 5] [79]. The options include oral morphine, oral oxycodone, hydromorphone, or transdermal fentanyl [Table 6].

Patients who require multiple doses of short-acting drugs daily should be switched to a long-acting formulation [Table 7] [80]. Morphine or hydromorphone may be preferred over oxycodone due to once-daily dosing. Transdermal fentanyl may be preferred over oral morphine in case of constipation.

#### 5.3.5. Opioid Dose Titration in Cancer Pain

Opioid dosage modification is consistently necessary to enhance the regimen and maintain its benefits over time. This necessitates a daily assessment of pain to evaluate adequate control of pain, severe side effects, and the patient’s need for more than 2–3 as-needed dosages each day. In cases of inadequate analgesia, the opioid dosage may be escalated until an optimal equilibrium between analgesia and adverse effects is achieved, or until the patient experiences unbearable and unmanageable side effects. The necessity for a relatively increased dosage (e.g., over 200 mg morphine) in a specific patient warrants meticulous revaluation. The total daily dosage should be augmented by 25–50%. The interval between dose escalations should be sufficiently prolonged to permit the attainment of a new steady state, which necessitates five to six half-lives, regardless of the administration route or medication. Patients experiencing severe pain require rapid dose increase. As the dosage of the fixed-schedule opioid regimen increases, the dosage of the PRN medication must similarly be augmented. Typically, the dosage of this short-acting medicine should be maintained between 5 and 15 percent of the total daily dosage [81,82].

In instances of inadequate pain relief, opioid rotation is implemented, defined as the transition from one opioid to another to obtain improved results. The initial dosage of the substitute medication should approximate the anticipated equianalgesic dose to avert withdrawal symptoms or inadvertent overdose [83].

For opioid conversion, calculate the total 24 h dose of the current opioid and convert to morphine oral equivalent (MME) by using equivalence ratios [Table 8]. The starting dose of all major opioids is equivalent to 30 mg of morphine per day orally.

#### 5.3.6. Breakthrough Cancer Pain

Breakthrough pain is a transitory, acute pain that occurs on a background of adequately controlled chronic pain. Breakthrough pain is severe in intensity, has a rapid onset, and usually lasts <30 min. Breakthrough pain is distinct from background pain, requires specific assessment, and should be treated with fast-acting interventions. Clinically significant breakthrough pain is usually managed by prescribing a rescue drug, which is generally a short-acting opioid such as immediate-release morphine, oxycodone, hydromorphone, or oxymorphone. The breakthrough doses are usually 10–15% of the total daily opioid dose, given every 2–4 h prn [84].

### 5.4. Opioid-Induced Hyperalgesia

Opioid-induced hyperalgesia (OIH) is a paradoxical phenomenon wherein patients utilizing opioids for analgesia exhibit heightened pain sensitivity, indicating that these drugs may exacerbate pain rather than alleviate it. The exact molecular mechanism of OIH remains unclear and exhibits significant variability in both basic research literature and clinical practice. It is commonly believed to arise from neuroplastic alterations in the peripheral and central nervous systems that induce sensitization of pronociceptive pathways. Numerous mechanisms have been hypothesized for OIH, with significant processes identified: the central glutamatergic system, spinal dynorphins, descending facilitation, hereditary factors, and reduced reuptake coupled with an amplified nociceptive response. The central glutamatergic system is regarded as the most prevalent option among these. Another concept includes NMDA receptors in OIH that involve activation, inhibition of the glutamate transporter system, facilitation of calcium-regulated intracellular protein kinase C, and interaction of brain systems related to pain and tolerance [85]. Recently thalamic T-channels have been postulated as a contributor to OIH that needs to be studied further [86]. Clinically, OIH may be suspected if individuals experience increased pain with escalating opioid doses, if pain radiates beyond its initial site, or if they exhibit atypical pain sensations such as allodynia. Management typically includes decreasing opioid dosage or transitioning to non-opioid options and, in certain instances, employing medications that target NMDA receptors. Identifying and addressing OIH is essential in pain management, since it exacerbates chronic pain and prolongs opioid treatment, highlighting the necessity for meticulous dosing and oversight in opioid utilization for pain relief [85].

### 5.5. Opioid Addiction and Reward Pathway

Opioid addiction arises from the powerful activation of the brain’s mesolimbic reward pathway, primarily involving the ventral tegmental area (VTA), nucleus accumbens (NAc), and prefrontal cortex. Opioids bind to MORs (key modulators in the reward circuitry) on inhibitory GABAergic neurons in the VTA, disinhibiting dopaminergic neurons and causing excessive dopamine release in the NAc, which produces intense euphoria and reinforcement. Chronic exposure leads to neuroadaptations, including receptor downregulation, altered intracellular signaling, and recruitment of stress pathways (such as the amygdala and locus coeruleus), contributing to tolerance, dependence, and withdrawal. Over time, natural rewards become less salient, while drug-seeking behavior is driven by both positive reinforcement (euphoria) and negative reinforcement (relief of withdrawal symptoms), perpetuating the cycle of addiction. Genetic variation plays an important role in modulating individual vulnerability to opioid response and misuse. One of the most studied variants is the OPRM1 A118G (rs1799971) polymorphism in the gene encoding the μ-opioid receptor, the primary target of most opioids. This single nucleotide substitution alters receptor binding and signaling, leading to measurable differences in analgesic efficacy, required opioid dose, and subjective reward responses. While some studies link the G allele to higher opioid use risk, results are inconsistent and drug-specific. Other genes (e.g., DRD2, COMT, CYP2D6, FKBP5) also contribute. Although not yet suitable for routine clinical screening, genetic factors help explain interindividual variability in opioid analgesia, tolerance, and addiction risk. Both the pain-relieving and harmful effects of opioids are mediated through μ-opioid receptors (MORs). Despite extensive research, no agent has yet been developed that provides effective analgesia without a risk of misuse. Current therapies for opioid addiction instead focus on preventing dependence, treating established dependence, and alleviating withdrawal symptoms [87].

### 5.6. Interventional Therapies for Cancer Pain

Interventional therapies may be helpful for cancer patients who are poorly responsive to opioid analgesics or develop intolerable side effects. Interventional therapies include invasive analgesic techniques such as injections, ablations, infusion therapies, neuromodulation, and some minimally invasive surgical techniques [88,89].

- Epidural and Intrathecal Analgesia: Opioids, local anesthetics, and adjuvant agents such as clonidine or ketamine can be delivered directly into the spinal pathways for the management of severe, refractory pain. In cancer patients with an anticipated survival of more than three months, long-term options such as an implanted intrathecal catheter with a subcutaneous infusion pump may provide sustained benefit. For patients with a more limited life expectancy, epidural therapy can be administered through an implanted catheter or port connected to an external patient-controlled analgesia (PCA) pump, offering effective pain relief without the need for permanent devices.
- Nerve Blocks: Nerve blocks involve the targeted injection of local anesthetics, steroids, or neurolytic agents to interrupt pain transmission along specific neural pathways. They are particularly useful for localized or regional cancer-related pain that does not respond adequately to systemic therapies. A celiac plexus block, most often employed in patients with pancreatic cancer, provides substantial relief of visceral abdominal pain, with reported success rates of 80–90%, and can significantly reduce opioid requirements. Intercostal nerve blocks are beneficial for pain arising from primary or metastatic tumors of the chest wall and pleura, as well as for post-mastectomy pain, implant-related discomfort, and post-herpetic intercostal neuralgia. By providing site-specific analgesia, nerve blocks not only improve pain control but also enhance functional status and quality of life, while minimizing systemic opioid exposure and associated adverse effects.
- Neuromodulation: Neuromodulation involves the use of electrical stimulation techniques to modify pain signaling pathways within the nervous system. It is typically considered for patients with neuropathic pain that is refractory to conventional pharmacologic or interventional approaches. The two principal modalities are spinal cord stimulation (SCS) and dorsal root ganglion (DRG) stimulation. SCS delivers electrical impulses to the dorsal columns of the spinal cord, thereby inhibiting pain transmission and reducing pain perception. It is particularly useful for widespread or diffuse neuropathic pain. DRG stimulation, on the other hand, provides more targeted stimulation at the dorsal root ganglion, offering precise pain relief for focal or regional neuropathic pain syndromes. Both techniques can improve analgesia, functional outcomes, and quality of life while reducing reliance on systemic analgesics, including opioids. Patient selection is critical, with careful consideration of pain type, prognosis, and functional goals, as well as a successful trial period prior to permanent implantation.
- Radiofrequency Ablation (RFA) and Cryoablation: Radiofrequency ablation is a minimally invasive procedure used for focal, refractory cancer-related pain, most commonly from bone metastases. Under image guidance, a probe delivers high-frequency alternating current, generating heat that induces thermal coagulation and necrosis of targeted nerve tissue or tumor cells. This interrupts pain transmission and can reduce tumor-related mass effect. RFA is especially effective for patients with localized severe bone pain unresponsive to systemic analgesics or radiation. In some cases, it is combined with cementoplasty (vertebroplasty/kyphoplasty) to provide structural stability. RFA has been shown to offer rapid, durable pain relief, improved mobility, and decreased opioid use, with relatively low complication rates (bleeding, infection, injury to adjacent structures). Cryoablation uses extreme cold (liquid nitrogen or argon gas) to create an ice ball at the tip of a probe, causing cell death and nerve disruption. It can be used for painful bone metastases as well as soft tissue tumors. Compared with RFA, cryoablation allows visualization of the ice ball under imaging, which helps precisely define the treatment area and protect surrounding structures. It is especially useful near critical nerves or vessels. Pain relief is typically significant, with some evidence suggesting better preservation of surrounding bone integrity compared to RFA. Potential risks include fracture, neuropathy, or local tissue damage, though overall complication rates remain low.
- Minimally Invasive Surgical Procedures: Vertebroplasty and kyphoplasty provide rapid pain relief and spinal stabilization in patients with painful vertebral metastases or compression fractures without neurologic compromise. Vertebroplasty involves direct injection of bone cement, while kyphoplasty uses balloon expansion before cement placement to restore height and reduce deformity. Both reduce analgesic needs and improve mobility, with low complication rates. Palliative Surgery may also be used for tumor debulking, relief of obstruction-related pain, or control of tumor bleeding improving symptom control and quality of life.
- Radiotherapy: Radiotherapy is a highly effective treatment for painful bone metastases and spinal cord compression, providing relief by reducing tumor burden, inflammation, and nerve compression. The standard regimens include 8 Gy in a single fraction for rapid palliation, or multi-fraction schedules such as 20 Gy in 5 fractions or 30 Gy in 10 fractions for patients with longer survival, offering improved local control and reduced retreatment rates. In cases of malignant spinal cord compression, 30 Gy in 10 fractions is commonly used to relieve pain and preserve neurological function.

### 5.7. Integrative Therapies for Cancer Pain

Integrative therapies such as acupuncture, hypnosis, massage, yoga and mindfulness-based therapies have shown efficacy in multimodal pain management in various randomized controlled trials. The recommendations by the Society for Integrative Oncology and ASCO on Integrative medicine for cancer pain management include [90]:

1. Acupuncture and yoga for aromatase inhibitor–related joint pain.
2. Acupuncture and guided imagery with progressive muscle relaxation for general cancer pain or musculoskeletal pain, reflexology or acupressure for pain during systemic cancer therapy, massage for chronic pain following breast cancer treatment, and hatha yoga for pain following treatment for breast or head and neck cancers.
3. For chemotherapy-induced peripheral neuropathy, acupuncture, reflexology or acupressure may be offered to patients experiencing chemotherapy-induced peripheral neuropathy.
4. For procedural or surgical pain, options include hypnosis, acupuncture, acupressure or music therapy.
5. Massage may be offered to patients experiencing pain during palliative and hospice care.

## 6. NCCN Clinical Practice Guidelines for Adult Cancer Pain, Version 2.2025

The NCCN Guidelines for Adult Cancer Pain emphasize systematic pain assessment at every encounter, integrating mechanism, severity, and psychosocial context. Management follows a stepwise approach: nonopioid agents, adjuvants, and bone-modifying therapies. Opioids remain central for moderate-to-severe pain, starting with short-acting formulations for titration, then long-acting agents for persistent pain, with breakthrough doses equal to 10–20% of the daily total. The guidelines stress risk mitigation, including opioid agreements, prescription drug monitoring program checks, urine drug testing in selected patients, constipation prophylaxis, and co-prescribing naloxone for high-risk individuals. If opioid use disorder is identified, treatment with buprenorphine or methadone should be integrated without compromising cancer pain care. For focal or refractory pain, interventional therapies, radiofrequency or cryoablation, vertebroplasty/kyphoplasty, and neuromodulation are recommended. Palliative radiotherapy remains highly effective for painful bone metastases and spinal cord compression. The guidelines also highlight the role of integrative and rehabilitative interventions including physical therapy, psychological support, and complementary approaches to optimize pain control and quality of life [91].

## 7. Biomarkers in Cancer Pain

Biomarkers in cancer pain are a rapidly evolving area of research aimed at identifying objective, measurable indicators that can predict, diagnose, or monitor pain in patients with cancer. These biomarkers can help personalize pain management strategies and improve outcomes [92,93]. In cancer pain, several types of biomarkers are under investigation:

- **Inflammatory Biomarkers:** Cytokines such as IL-6, IL-1β, and TNF-α are elevated in patients with cancer pain, especially those with bone metastases or neuropathic pain. C-reactive protein correlates with systemic inflammation and the severity of pain.
- **Genetic and Epigenetic Biomarkers:** Polymorphisms in genes like OPRM1 (μ-opioid receptor), COMT (catechol-O-methyltransferase), and CYP2D6 can influence opioid response, metabolism, and susceptibility to pain. Epigenetic changes (e.g., DNA methylation in pain-related genes) may modulate individual pain perception [93].
- **Neurotransmitters and Neuromodulators:** Substance P and calcitonin gene-related peptide are involved in nociceptive transmission and may be elevated in chronic and neuropathic cancer pain. Brain-derived neurotrophic factor is implicated in pain sensitization and the development of chronicity [93].
- **Neuroimaging Biomarkers:** Functional MRI (fMRI) and PET scans can detect altered activity in pain-processing brain regions, potentially serving as objective pain biomarkers in the future [93].
- **Metabolomic and Microbiome Biomarkers:** Metabolomic changes, such as high lactate levels, which are linked to muscle pain, may help understand the metabolic changes in pain disorders. Gut flora compositions may help identify the links between gut health and chronic pain [93].

## 8. Personalized Approach to Cancer Pain Management: Integrating Precision Medicine

While WHO guidelines provide effective pain relief for about 75% of cancer patients, they often fall short in addressing the complex, multidimensional nature of cancer pain [94]. Cancer pain is highly individualized, influenced by diverse pain mechanisms, tumor characteristics, and patient-specific factors. Precision medicine tailors pain assessment and treatment to each patient’s unique biological and genetic profile, while the personalized approach tailors care for the whole patient. Integrating both offers the most effective, equitable, and compassionate cancer pain management [95,96,97].

Therefore, a multimodal strategy is essential, incorporating:

1. **Pain Mechanisms, Phenotypes, and Biomarkers:** Cancer pain can be nociceptive, neuropathic, or mixed. For instance, bone metastases typically cause inflammatory nociceptive pain, whereas nerve compression results in neuropathic pain, which may be less responsive to opioids. Many patients experience overlapping pain mechanisms, necessitating a combination of opioids and adjuvant therapies. Precision pain medicine involves systematically identifying the dominant pain phenotype (nociceptive, neuropathic, or mixed) in each patient, which guides the choice of pharmacologic and non-pharmacologic therapies. Emerging biomarkers are being explored to define pain phenotypes better and guide individualized treatment strategies [92,93].
2. **Tumor Type, Stage, and Treatment:** Pain varies by cancer type, location, stage, and treatment received. Advanced malignancies (such as pancreatic or head and neck cancers) and interventions like surgery, chemotherapy, or radiation often intensify pain, requiring both pharmacologic and interventional approaches tailored to the patient’s clinical context.
3. **Patient-Specific Factors:** Individual characteristics, including age, genetics, organ function, prior opioid exposure, and psychosocial factors, significantly influence pain perception and management. Older adults may have altered drug metabolism and heightened sensitivity to opioids, necessitating careful dosing and monitoring. Genetic variations, such as in CYP2D6, COMT, and OPRM1, can influence how patients metabolize and respond to analgesics, especially opioids. Testing for these variants may help clinicians select the most effective drugs and dosages, reducing side effects and improving pain control [93,97]. Comorbidities like renal or hepatic dysfunction require thoughtful opioid selection and dose adjustments. Psychological factors (depression, anxiety), substance use, cultural background, language barriers, and health literacy all impact pain expression, reporting, and treatment adherence. Addressing these factors is crucial for equitable and adequate pain control.

### Framework for Personalized Cancer Pain Management

We propose a five-domain framework to address the complexity of cancer pain [Table 9]:

- Biological—Pain mechanism, tumor type, biomarkers
- Pharmacologic—Opioid sensitivity, prior exposure, drug metabolism
- Psychological—Mental health, coping skills, support systems
- Sociocultural—Language, beliefs, stigma, access to care
- Functional—Performance status, Caregiver availability, Ability to manage complex regimens

Multidisciplinary teams should assess these domains and construct individualized care plans.

## 9. New Advances in Cancer Pain Management

### 9.1. Targeted Therapies

#### 9.1.1. Protein Kinase Inhibitors

Various protein kinases, such as tropomyosin-related kinases (Trks) including TrkA, TrkB and TrkC, protein kinases A, G, and C, p38 MAPK (p38 mitogen-activated protein kinase), ERK (extracellular signal-regulated kinase), PKG (protein kinase G), mTOR (mammalian target of rapamycin), MNKs (MAPK-interacting kinases), cyclin-dependent kinases (CDKs) such as CDK5 have emerged as potential targets that directly affect pain signaling pathways [98,99]. The role of nerve growth factor (NGF) and its receptor TrkA in chronic pain is well established, and treatments targeting the NGF-TrkA pathway have shown considerable analgesic efficacy [100]. Tanezumab, a monoclonal antibody targeting NGF, has shown efficacy in treating pain associated with bone metastases [101]. The advancement of innovative protein kinase inhibitors for the therapy of cancer-related pain is complex and necessitates a more profound comprehension of their function in pain mechanisms.

#### 9.1.2. Ion Channel Inhibitors

Ion channel inhibitors have demonstrated potential in the treatment of neuropathic pain and cancer-related bone pain. Voltage-gated ion channels, such as sodium (NaV), calcium (CaV), and potassium (KV) channels, are crucial in regulating neuronal excitability and the transmission of pain signals after nerve injury. NaV channels are significant in the establishment and sustenance of neuropathic pain. Research in both animals and humans has confirmed that sodium channels, particularly NaV1.7, NaV1.8, and NaV1.9, are effective targets for pain management. VX-548, a selective NaV1.8 inhibitor, has received FDA approval for the management of moderate to severe acute pain. It is under investigation for its efficacy in managing neuropathic pain, presenting a non-opioid option with a benign side effect profile and no risk of addiction. CaV2.1 (P/Q-type) and CaV2.2 (N-type) channels are crucial for neuronal excitability, synaptic transmission, and pain signaling. Ziconotide has been recognized as an effective antagonist of CaV2.2 channels for the management of severe persistent neuropathic pain. C2230 is an innovative inhibitor of CaV2.2 channels, noted for its analgesic efficacy in several pain types. KV1.2 channels have been recognized as molecular contributors to the pathophysiology of neuropathic pain among potassium (KV) channels; nevertheless, they have not yet been utilized as a therapeutic target. The complex nature of neuropathic pain and the wide variety of ion channels present enormous challenges in the study of voltage-gated ion channels within this context [102].

Transient Receptor Potential (TRP) ion channels have been demonstrated to influence pain perception associated with nociceptive and neuropathic pain, perhaps contributing to the pathophysiology of CIBP. TRP channels are categorized into: TRPC (canonical), TRPA (Ankyrin), TRPM (melastatins), TRPML (mucolipins), TRPP (polycystins), TRPV (vanilloids), and TRPN (no mechanoreceptor potential C channels), each exhibiting distinct features. TRP ion channels are associated with bone metabolism and several illnesses, such as osteoporosis and bone metastases. TRPV1 and TRPV2 are implicated in the progression of multiple myeloma, whereas TRPM7 facilitates disease dispersion. TRPV1, commonly referred to as the “capsaicin receptor,” is characterized by its sensitivity to the vanilloid compound capsaicin. TRPV1 was initially evaluated in bone cancer pain in 2005, revealing that most sensory neurons in malignant bone expressed TRPV1, and subcutaneous treatment of its antagonist, JNJ-17203212, mitigated pain-related behaviors. TRPV1 antagonists diminished nociceptive sensitivity in preclinical pain models, particularly with CIBP, exhibiting diversity in analgesic efficacy, potentially attributable to changes in their pharmacological characteristics. Resiniferatoxin, a highly powerful counterpart of capsaicin, functions as a TRPV1 agonist and has been evaluated in patients with advanced cancer and refractory pain, yielding a good response. Numerous other TRP channel modulators are currently under investigation and may represent a promising area of research in CIBP [103].

### 9.2. Artificial Intelligence (AI) and Digital Health

Artificial intelligence is progressively used in healthcare to improve patient safety in multiple processes, including pain assessment. Conventional pain assessment methods may be subjective and affected by several factors, resulting in a misconception of pain intensity.

AI-driven camera-based methods have emerged as alternatives to conventional methods for PROs, eliminating the need for physical contact. They are particularly beneficial for patients with difficulty communicating verbally, such as infants or those with dementia. AI for face recognition can capture facial expressions to recognize pain. These camera-based approaches can be integrated into pain management protocols for cancer patients in inpatient and home care settings. Contact-Sensor approaches, such as electrodermal activity, electrocardiogram, and electroencephalography, may be used for pain evaluation. Other methods include incorporating AI algorithms in audio events and voice recognition for pain assessment. Advances in AI are beginning to transform the treatment and relief of cancer-related pain as we navigate the field of oncology [104].

## 10. Challenges in Advancing Precision and Personalized Cancer Pain

While precision and personalized medicine offer promising avenues for improving cancer pain management, several critical challenges must be addressed to realize their potential fully:

1. **Tumor and Patient Heterogeneity**

The complex interplay between tumor biology, pain mechanisms, and individual patient variability makes it challenging to predict pain experiences and tailor interventions effectively. Differences in pain phenotypes and underlying pathophysiology contribute to inconsistent treatment outcomes [105].

2. **Limited Biomarkers and Pharmacogenomic Tools**

The lack of validated biomarkers and genetic predictors of pain perception and analgesic response hinders the development of personalized analgesic regimens. Progress in pharmacogenomics remains slow, limiting clinicians’ ability to optimize treatment based on individual genetic profiles [93].

3. **Data Complexity and Integration**

Leveraging high-throughput genomic and clinical data requires sophisticated infrastructure and analytic frameworks. Challenges in standardization, data sharing, and interoperability limit the practical use of precision tools in clinical settings [106].

4. **Access and Equity**

The high cost of genomic testing and precision diagnostics, along with geographic and systemic barriers, restricts access for many patients, particularly those in low-resource or underserved settings, exacerbating existing disparities in pain management and cancer care [106].

5. **Digital Health Integration**

Cancer pain management is complex and often unfolds within the context of competing personal goals and limited resources. Digital health technologies, such as mobile apps and remote symptom monitoring platforms, offer opportunities to personalize pain tracking and improve patient–clinician communication. However, the challenge lies in ensuring that these tools truly add value without overburdening patients, caregivers, or clinicians. Solutions must be intuitive, adaptive, and seamlessly integrated into care workflows to be effective and equitable [107].

## 11. Conclusions

Cancer pain management has evolved significantly, from historical under-treatment to modern multimodal and targeted approaches. While opioids remain a cornerstone, newer pharmacological and interventional strategies, including targeted therapies, neuromodulation, and integrative therapies, offer promising alternatives. The integration of AI-driven pain assessment and digital health tools further refines individualized treatment strategies. Central to this evolution is the recognition that cancer pain is complex and deeply personal, requiring care tailored to each patient’s unique biology, psychology, and sociocultural factors. While advances in precision and personalized medicine are transforming cancer pain management, challenges related to tumor and patient heterogeneity, biomarker validation, data complexity, access, clinical implementation, safety, and inclusivity must be addressed to realize the full potential of these innovations.

## Figures and Tables

**Table 1:** WHO Analgesic Ladder.

Step 1	Mild Pain	Non-Opioid Analgesics Such as Nonsteroidal Anti-Inflammatory Drugs and Acetaminophen.
Step 2	Moderate pain	Weak opioids like codeine or tramadol are used when pain persists or increases.
Step 3	Severe pain	Potent opioids such as morphine, oxycodone, or fentanyl, often in combination with non-opioids and adjuvant therapies for additive benefits.

**Table 2:** WHO recommendations on the proper use of analgesics include.

Oral Administration of Analgesics	Whenever Possible, the Oral Form Should Be Preferred.
Analgesics should be given at regular intervals.	Prescribe the dosage to be taken at regular intervals based on the patient’s level of pain. The dosage of medication should be adjusted until the patient is comfortable and experiencing relief from their symptoms.
Analgesics should be prescribed according to pain intensity.	Pain medications should be prescribed after a proper assessment of the pain using pain scales.
Dosing pain medication should be adapted to the individual.	There is no standardized dosage for treating pain. The correct dosage will provide adequate pain relief.
Analgesics should be prescribed with meticulous attention to detail.	The regularity of analgesic administration is crucial for effective pain treatment. Once the distribution of medication over a day is established, it is ideal to provide the patient with a written personal program. In this way, the patient, his family, and medical staff will all have the necessary information about when and how to administer the medications.

**Table 4:** ASCO Recommendations on Opioid Use for Cancer Pain.

Questions	Recommendations
Initiation of opioids	All patients with cancer having moderate-to-severe pain should be offered opioids unless contraindicated. Healthcare providers, patients, and caregivers should engage in discussions regarding objectives pertaining to functional results, possible adverse effects, and the significance of compliance with the prescribed treatment plan.
Choice of opioids	Any opioid approved by the FDA or other regulatory bodies for analgesic purposes. The selection is determined by parameters such as pharmacokinetic characteristics, including bioavailability, administration method, half-life, neurotoxicity, and the cost of various medications.
Initial dose and titration	Begin with the lowest effective dose of short-acting opioids and PRN (as needed). Early assessment and frequent titration are essential to achieve optimal pain control. Patients may persist with non-opioid medications after the commencement of opioids if they offer supplementary analgesia and are not contraindicated. Doses should be augmented by 25–50%, while accounting for patient-specific characteristics such as frailty, comorbidities, and organ function.
Opioids in renal or hepatic dysfunction	Morphine, meperidine, codeine, and tramadol should be avoided unless no alternatives are available. Methadone can be administered safely in cases with renal impairment. Fentanyl, oxycodone, and hydromorphone must be meticulously titrated and regularly checked for deleterious effects.
Opioids for Breakthrough Pain	For patients on continuous opioid therapy, short-acting opioids should be administered at a dosage of 5–20% of the total daily morphine equivalent for the management of breakthrough pain.
Opioid Rotation	Opioid rotation should be considered for individuals experiencing insufficient pain relief, excessive adverse effects, logistical or financial issues, or difficulties with the method of opioid delivery or absorption.

**Table 5:** Short-Acting (Immediate-Release IR) Opioids.

Opioid	Route	Typical Duration
Morphine IR	Oral	3–4 h
Oxycodone IR	Oral	3–5 h
Hydromorphone IR	Oral, IV	2–4 h
Tramadol IR	Oral	4–6 h
Codeine	Oral	4–6 h
Fentanyl IV	IV	30–60 min

**Table 6:** Initial Dosing in Opioid-Naïve Patients.

Drug	Typical Starting Dose (PO)	Notes
Morphine IR	5–10 mg every 3–4 h	Gold Standard
Oxycodone IR	2.5–5 mg every 4 h	Slightly more potent than morphine
Hydromorphone	1–2 mg every 3–4 h	Potent can be used in renal dysfunction
Methadone	2.5 mg every 8–12 h	Long acting: expert titration needed.
Fentanyl	IV or patch; 12 mcg/h every 72 h if tolerant	High potency; patch if a stable dose is achieved.

**Table 7:** Long-Acting (Extended-Release ER) Opioids.

Opioid	Route	Typical Duration
Morphine ER (MS Contin)	Oral	8–12 h
Oxycodone ER (OxyContin)	Oral	12 h
Fentanyl patch	Transdermal	72 h
Methadone	Oral	6–12 h
Tapentadol ER	Oral	12 h
Hydromorphone ER	Oral	24 h

**Table 8:** Oral Morphine Milligram Equivalent (MME) Table.

Opioid	Route	Conversion to Oral Morphine (MME)
Morphine	PO	30 mg = 30 mg
Morphine IV	IV	10 mg = 30 mg
Oxycodone	PO	20 mg = 30 mg
Hydromorphone	PO	7.5 mg = 30 mg
Hydromorphone	IV	1.5 mg = 30 mg
Oxymorphone	PO	10 mg = 30 mg
Fentanyl	Transdermal	12 mcg/h = 30–45 mg/day
Methadone	PO	Highly variable; if MME < 100, then ratio = 1:3–4 If MME 100–300, then ratio = 1:5–10 If MME > 300, then ratio = 1:12–20
Tapentadol	PO	100 mg = 30 mg
Codeine	PO	200 mg = 30 mg
Tramadol	PO	120 mg = 30 mg

**Table 9:** Framework for Personalized Cancer Pain Management.

Domain	Patient-Specific Factors	Implications for Pain Management
Biological	- Tumor type and location - Pain mechanism (nociceptive, neuropathic, visceral, mixed) - Disease stage - Genetics and biomarkers	- Select mechanism-targeted agents (e.g., opioids, adjuvants) - Consider early use of interventional techniques (e.g., nerve blocks) - Monitor evolving pain syndromes with disease progression. - Incorporate biomarkers.
Pharmacologic	- Age and organ function (renal, hepatic) - Opioid tolerance or prior exposure - Drug–drug interactions (especially in polypharmacy)	- Adjust dosing and opioid choice based on metabolism - Rotate opioids in case of tolerance or side effects - Use extended release or adjuvants judiciously in the elderly
Psychological	- Depression, anxiety, PTSD - Pain catastrophe - Cognitive status	- Incorporate psychological support and cognitive behavioral therapy (CBT) - Use integrative approaches (e.g., mindfulness, music therapy, acupuncture) - Screen for and treat psychiatric illnesses.
Sociocultural	- Language barriers - Cultural beliefs about pain/opioids - Health literacy - Access to care and insurance - Equity focus	- Use interpreters and culturally sensitive communication - Educate patients and families about pain control and opioid safety - Address stigma and fear associated with opioid use
Functional	- Performance status - Caregiver availability - Ability to manage complex regimens	- Simplify dosing regimens - Engage caregivers in pain monitoring - Consider hospice or home palliative services as needed

## Data Availability

No new data were created or analyzed in this study. Data sharing is not applicable to this article.

## Abbreviations

WHO	World Health Organization
CIBP	Cancer-induced bone pain
RANKL	Receptor activators of nuclear factor-kappa-B ligand
TNF	Tumor necrosis factor
IL	Interleukin
ICD	International Classification of Diseases
IASP	International Association for the Study of Pain
PROs	Patient-Reported Outcomes
VAS	Visual Analog Scale
NRS	Numerical Rating Scale
MPQ	McGill Pain Questionnaire
BPI	Brief Pain Inventory
AIDS	Acquired immunodeficiency syndrome
NSAIDS	Nonsteroidal Anti-Inflammatory Drugs
COX	cyclooxygenase
SNRIs	serotonin-norepinephrine reuptake inhibitors.
TCAs	tricyclic antidepressants
CB	Cannabinoids
THC	tetrahydrocannabinol
CBD	cannabidiol
NMDA	N-methyl-D-aspartate
SREs	skeletal-related events
PEA	Palmitoylethanolamide
MORs	mu opioid receptors
IV	intravenous
SC	subcutaneous
ASCO	American Society of Clinical Oncology
IR	Immediate-Release
ER	Extended-Release
PRN	Pro re nata “as needed”
MME	morphine oral equivalent
OIH	Opioid-induced hyperalgesia
PCA	patient-controlled analgesia
SCS	spinal cord stimulation
DRG	dorsal root ganglion
RFA	Radiofrequency ablation
OPRM1	μ-opioid receptor
COMT	Catechol-O-methyltransferase
PTSD	Post traumatic stress disorder
CBT	cognitive behavioral therapy
Trks	tropomyosin-related kinases
MAPK	Mitogen-activated protein kinase
ERK	Extracellular signal-regulated kinase
PKG	Protein kinase G
Mtor	mammalian target of rapamycin
MNKs	MAPK-interacting kinases
CDKs	Cyclin-dependent kinases
NGF	Nerve growth factor
TRP	Transient Receptor Potential
AI	Artificial Intelligence
NCCN	National Comprehensive Cancer Network

## References

[1] Snijders R.A.H., Brom L., Theunissen M., van den Beuken-van Everdingen M.H.J. (2023). Update on Prevalence of Pain in Patients with Cancer 2022: A Systematic Literature Review and Meta-Analysis. Cancers.

[2] Mestdagh F., Steyaert A., Lavand’homme P. (2023). Cancer Pain Management: A Narrative Review of Current Concepts, Strategies, and Techniques. Curr. Oncol..

[3] Evenepoel M., Haenen V., De Baerdemaecker T., Meeus M., Devoogdt N., Dams L., Van Dijck S., Van der Gucht E., De Groef A. (2022). Pain Prevalence During Cancer Treatment: A Systematic Review and Meta-Analysis. J. Pain Symptom Manag..

[4] Kroenke K., Theobald D., Wu J., Loza J.K., Carpenter J.S., Tu W. (2010). The association of depression and pain with health-related quality of life, disability, and health care use in cancer patients. J. Pain Symptom Manag..

[5] Ośmiałowska E., Misiąg W., Chabowski M., Jankowska-Polańska B. (2021). Coping Strategies, Pain, and Quality of Life in Patients with Breast Cancer. J. Clin. Med..

[6] Pérez C., Ochoa D., Sánchez N., Ballesteros A.I., Santidrián S., López I., Mondéjar R., Carnaval T., Villoria J., Colomer R. (2024). Pain in Long-Term Cancer Survivors: Prevalence and Impact in a Cohort Composed Mostly of Breast Cancer Survivors. Cancers.

[7] Hamilton G.R., Baskett T.F. (2000). In the arms of Morpheus the development of morphine for postoperative pain relief. Can. J. Anaesth..

[8] Berridge V. (2009). Heroin prescription and history. N. Engl. J. Med..

[9] Brook K., Bennett J., Desai S.P. (2017). The Chemical History of Morphine: An 8000-year Journey, from Resin to de-novo Synthesis. J. Anesth. Hist..

[10] Laing R., Donnelly C.A. (2024). Evolution of an epidemic: Understanding the opioid epidemic in the United States and the impact of the COVID-19 pandemic on opioid-related mortality. PLoS ONE.

[11] Paice J.A. (2018). Cancer pain management and the opioid crisis in America: How to preserve hard-earned gains in improving the quality of cancer pain management. Cancer.

[12] Ventafridda V., Saita L., Ripamonti C., De Conno F. (1985). WHO guidelines for the use of analgesics in cancer pain. Int. J. Tissue React..

[13] Wilson J., Stack C., Hester J. (2014). Recent advances in cancer pain management. F1000Prime Rep..

[14] Kettyle G. (2023). Multidisciplinary Approach to Cancer Pain Management. Ulst. Med. J..

[15] Falk S., Bannister K., Dickenson A.H. (2014). Cancer pain physiology. Br. J. Pain.

[16] Pahuta M., Laufer I., Lo S.-F.L., Boriani S., Fisher C., Dea N., Weber M.H., Chou D., Sahgal A., Rhines L. (2025). Defining Spine Cancer Pain Syndromes: A Systematic Review and Proposed Terminology. Glob. Spine J..

[17] Haroun R., Wood J.N., Sikandar S. (2023). Mechanisms of cancer pain. Front. Pain Res..

[18] Coleman R.E. (2006). Clinical features of metastatic bone disease and risk of skeletal morbidity. Clin. Cancer Res..

[19] Foley K.M. (2004). Treatment of cancer-related pain. J. Natl. Cancer Inst. Monogr..

[20] Lipton A. (2004). Pathophysiology of bone metastases: How this knowledge may lead to therapeutic intervention. J. Support. Oncol..

[21] Jones D.H., Nakashima T., Sanchez O.H., Kozieradzki I., Komarova S.V., Sarosi I., Morony S., Rubin E., Sarao R., Hojilla C.V. (2006). Regulation of cancer cell migration and bone metastasis by RANKL. Nature.

[22] Akech J., Wixted J.J., Bedard K., van der Deen M., Hussain S., Guise T.A., van Wijnen A.J., Stein J.L., Languino L.R., Altieri D.C. (2010). Runx2 association with progression of prostate cancer in patients: Mechanisms mediating bone osteolysis and osteoblastic metastatic lesions. Oncogene.

[23] Eliasson P., Jönsson J.I. (2010). The hematopoietic stem cell niche: Low in oxygen but a nice place to be. J. Cell. Physiol..

[24] Regan J.M., Peng P. (2000). Neurophysiology of cancer pain. Cancer Control.

[25] Sikandar S., Dickenson A.H. (2012). Visceral pain—The ins and outs, the ups and downs. Curr. Opin. Support. Palliat. Care.

[26] Wang L., Xu H., Ge Y., Zhu H., Yu D., Yu W., Lu Z. (2017). Establishment of a murine pancreatic cancer pain model and microarray analysis of pain-associated genes in the spinal cord dorsal horn. Mol. Med. Rep..

[27] Lam D.K., Schmidt B.L. (2010). Serine proteases and protease-activated receptor 2-dependent allodynia: A novel cancer pain pathway. Pain.

[28] Edwards H.L., Mulvey M.R., Bennett M.I. (2019). Cancer-Related Neuropathic Pain. Cancers.

[29] Yoon S.Y., Oh J. (2018). Neuropathic cancer pain: Prevalence, pathophysiology, and management. Korean J. Intern. Med..

[30] Rayment C., Hjermstad M.J., Aass N., Kaasa S., Caraceni A., Strasser F., Heitzer E., Fainsinger R., Bennett M.I., On behalf of the European Palliative Care Research Collaborative (EPCRC) (2013). Neuropathic cancer pain: Prevalence, severity, analgesics and impact from the European Palliative Care Research Collaborative-Computerised Symptom Assessment study. Palliat. Med..

[31] Delanian S., Lefaix J.L., Pradat P.F. (2012). Radiation-induced neuropathy in cancer survivors. Radiother. Oncol..

[32] Portenoy R.K., Ahmed E. (2018). Cancer Pain Syndromes. Hematol. Oncol. Clin. N. Am..

[33] Paice J.A., Portenoy R., Lacchetti C., Campbell T., Cheville A., Citron M., Constine L.S., Cooper A., Glare P., Keefe F. (2016). Management of Chronic Pain in Survivors of Adult Cancers: American Society of Clinical Oncology Clinical Practice Guideline. J. Clin. Oncol..

[34] Bennett M.I., Kaasa S., Barke A., Korwisi B., Rief W., Treede R.D., IASP Taskforce for the Classification of Chronic Pain (2019). The IASP classification of chronic pain for ICD-11: Chronic cancer-related pain. Pain.

[35] Fink R.M., Gallagher E. (2019). Cancer Pain Assessment and Measurement. Semin. Oncol. Nurs..

[36] Jacobsen R., Liubarskiene Z., Møldrup C., Christrup L., Sjøgren P., Samsanaviciene J. (2009). Barriers to cancer pain management: A review of empirical research. Medicina.

[37] Hjermstad M.J., Fayers P.M., Haugen D.F., Caraceni A., Hanks G.W., Loge J.H., Fainsinger R., Aass N., Kaasa S., European Palliative Care Research Collaborative (EPCRC) (2011). Studies comparing Numerical Rating Scales, Verbal Rating Scales, and Visual Analogue Scales for assessment of pain intensity in adults: A systematic literature review. J. Pain Symptom Manag..

[38] Waldman S.D. (2011). Pain Management.

[39] Main C.J. (2016). Pain Assessment in Context: A State of the Science Review of the McGill Pain Questionnaire 40 years on. Pain.

[40] Cleeland C.S., Ryan K.M. (1994). Pain assessment: Global use of the Brief Pain Inventory. Ann. Acad. Med. Singap..

[41] Kaasalainen S. (2007). Pain assessment in older adults with dementia: Using behavioral observation methods in clinical practice. J. Gerontol. Nurs..

[42] van Herk R., van Dijk M., Baar F.P., Tibboel D., de Wit R. (2007). Observation scales for pain assessment in older adults with cognitive impairments or communication difficulties. Nurs. Res..

[43] Lemaire A., George B., Maindet C., Burnod A., Allano G., Minello C. (2019). Opening up disruptive ways of management in cancer pain: The concept of multimorphic pain. Support. Care Cancer.

[44] Dworkin R.H., O’Connor A.B., Backonja M., Farrar J.T., Finnerup N.B., Jensen T.S., Kalso E.A., Loeser J.D., Miaskowski C., Nurmikko T.J. (2007). Pharmacologic management of neuropathic pain: Evidence-based recommendations. Pain.

[45] Vargas-Schaffer G. (2010). Is the WHO analgesic ladder still valid? Twenty-four years of experience. Can. Fam. Physician.

[46] Gómez-Cortéz M.D., Rodríguez-Huertas F. (2000). Reevaluación del segundo escalón de la escalera analgésica de la OMS. Rev. Soc. Esp. Dolor.

[47] Vargas-Schaffer G., Morales Pola E.A. (2002). Tratamiento del dolor oncologico y cuidados paliativos en pediatría. Manual de Clinica del Dolor y Cuidados Paliativos.

[48] Vargas-Schaffer G., Godoy D. (2004). Conceptos básicos del uso de opioides en el tratamiento del dolor oncológico. Rev. Venez. Oncol..

[49] Bruera E., Palmer J.L., Bosnjak S., Rico M.A., Moyano J., Sweeney C., Strasser F., Willey J., Bertolino M., Mathias C. (2004). Methadone versus morphine as a first-line strong opioid for cancer pain: A randomized double-blind study. J. Clin. Oncol..

[50] Clark A.J., Lynch M.E., Ware M., Beaulieu P., McGilveray I.J., Gourlay D. (2005). Guidelines for the use of cannabinoid compounds in chronic pain. Pain Res. Manag..

[51] Moulin D.E., Clark A.J., Gilron I., Ware M.A., Watson C.P., Sessle B.J., Coderre T., Morley-Forster P., Stinson J., Boulanger A. (2007). Pharmacological management of chronic neuropathic pain—Consensus statement and guidelines from the Canadian Pain Society. Pain Res. Manag..

[52] Mercadante S. (2001). The use of anti-inflammatory drugs in cancer pain. Cancer Treat. Rev..

[53] Howard A., Brant J.M. (2019). Pharmacologic Management of Cancer Pain. Semin. Oncol. Nurs..

[54] Kane C.M., Mulvey M.R., Wright S., Craigs C., Wright J.M., Bennett M.I. (2018). Opioids combined with antidepressants or antiepileptic drugs for cancer pain: Systematic review and meta-analysis. Palliat. Med..

[55] Leppert W., Buss T. (2012). The role of corticosteroids in the treatment of pain in cancer patients. Curr. Pain Headache Rep..

[56] Nersesyan H., Slavin K.V. (2007). Current approach to cancer pain management: Availability and implications of different treatment options. Ther. Clin. Risk Manag..

[57] Jose A., Thomas L., Baburaj G., Munisamy M., Rao M. (2020). Cannabinoids as an Alternative Option for Conventional Analgesics in Cancer Pain Management: A Pharmacogenomics Perspective. Indian J. Palliat. Care.

[58] Body J.J., Mancini I. (2002). Bisphosphonates for cancer patients: Why, how, and when?. Support. Care Cancer.

[59] Wong R., Wiffen P.J. (2002). Bisphosphonates for the relief of pain secondary to bone metastases. Cochrane Database Syst. Rev..

[60] Hamdy N.A. (2008). Denosumab: RANKL inhibition in the management of bone loss. Drugs Today.

[61] Tsourdi E., Rachner T.D., Rauner M., Hamann C., Hofbauer L.C. (2011). Denosumab for bone diseases: Translating bone biology into targeted therapy. Eur. J. Endocrinol..

[62] Choi E., Nahm F.S., Han W.K., Lee P.B., Jo J. (2020). Topical agents: A thoughtful choice for multimodal analgesia. Korean J. Anesthesiol..

[63] Davis M.P. (2024). Novel drug treatments for pain in advanced cancer and serious illness: A focus on neuropathic pain and chemotherapy-induced peripheral neuropathy. Palliat. Care Soc. Pract..

[64] Sah D., Shoffel-Havakuk H., Tsur N., Uhelski M.L., Gottumukkala V., Cata J.P. (2024). Opioids and Cancer: Current Understanding and Clinical Considerations. Curr. Oncol..

[65] Portenoy R.K., Hagen N.A. (1990). Breakthrough pain: Definition, prevalence and characteristics. Pain.

[66] Boland J.W. (2023). Tapentadol for the management of cancer pain in adults: An update. Curr. Opin. Support. Palliat. Care.

[67] Fallon M., Giusti R., Aielli F., Hoskin P., Rolke R., Sharma M., Ripamonti C.I., ESMO Guidelines Committee (2018). Management of cancer pain in adult patients: ESMO Clinical Practice Guidelines. Ann. Oncol..

[68] Leppert W. (2009). Tramadol as an analgesic for mild to moderate cancer pain. Pharmacol. Rep..

[69] Arbaiza D., Vidal O. (2007). Tramadol in the treatment of neuropathic cancer pain: A double-blind, placebo-controlled study. Clin. Drug Investig..

[70] Straube C., Derry S., Jackson K.C., Wiffen P.J., Bell R.F., Strassels S., Straube S. (2014). Codeine, alone and with paracetamol (acetaminophen), for cancer pain. Cochrane Database Syst. Rev..

[71] Leppert W. (2010). Dihydrocodeine as an opioid analgesic for the treatment of moderate to severe chronic pain. Curr. Drug Metab..

[72] Penson R.T., Joel S.P., Gloyne A., Clark S., Slevin M.L. (2005). Morphine analgesia in cancer pain: Role of the glucuronides. J. Opioid Manag..

[73] Schmidt-Hansen M., Bennett M.I., Arnold S., Bromham N., Hilgart J.S. (2022). Oxycodone for cancer-related pain. Cochrane Database Syst. Rev..

[74] Quigley C., Wiffen P. (2003). A systematic review of hydromorphone in acute and chronic pain. J. Pain Symptom Manag..

[75] Hadley G., Derry S., Moore R.A., Wiffen P.J. (2013). Transdermal fentanyl for cancer pain. Cochrane Database Syst. Rev..

[76] Mammana G., Bertolino M., Bruera E., Orellana F., Vega F., Peirano G., Bunge S., Armesto A., Dran G. (2021). First-line methadone for cancer pain: Titration time analysis. Support. Care Cancer.

[77] Samet J.H., Botticelli M., Bharel M. (2018). Methadone in Primary Care—One Small Step for Congress, One Giant Leap for Addiction Treatment. N. Engl. J. Med..

[78] Paice J.A., Bohlke K., Barton D., Craig D.S., El-Jawahri A., Hershman D.L., Kong L.R., Kurita G.P., LeBlanc T.W., Mercadante S. (2023). Use of Opioids for Adults With Pain From Cancer or Cancer Treatment: ASCO Guideline. J. Clin. Oncol..

[79] Huang R., Jiang L., Cao Y., Liu H., Ping M., Li W., Xu Y., Ning J., Chen Y., Wang X. (2019). Comparative Efficacy of Therapeutics for Chronic Cancer Pain: A Bayesian Network Meta-Analysis. J. Clin. Oncol..

[80] Chou R., Clark E., Helfand M. (2003). Comparative efficacy and safety of long-acting oral opioids for chronic non-cancer pain: A systematic review. J. Pain Symptom Manag..

[81] Jost L., Roila F., ESMO Guidelines Working Group (2008). Management of cancer pain: ESMO clinical recommendations. Ann. Oncol..

[82] Cormie P.J., Nairn M., Welsh J., Guideline Development Group (2008). Control of pain in adults with cancer: Summary of SIGN guidelines. BMJ.

[83] Reddy A., Yennurajalingam S., Pulivarthi K., Palla S.L., Wang X., Kwon J.H., Frisbee-Hume S., Bruera E. (2013). Frequency, outcome, and predictors of success within 6 weeks of an opioid rotation among outpatients with cancer receiving strong opioids. Oncologist.

[84] Caraceni A., Martini C., Zecca E., Portenoy R.K., Working Group of an IASP Task Force on Cancer Pain (2004). Breakthrough pain characteristics and syndromes in patients with cancer pain. An international survey. Palliat. Med..

[85] Lee M., Silverman S.M., Hansen H., Patel V.B., Manchikanti L. (2011). A comprehensive review of opioid-induced hyperalgesia. Pain Physician.

[86] Todorovic S.M. (2022). Opioid-induced hyperalgesia: Are thalamic T-type calcium channels treatment targets?. J. Clin. Investig..

[87] Zhang J.J., Song C.G., Dai J.M., Li L., Yang X.M., Chen Z.N. (2022). Mechanism of opioid addiction and its intervention therapy: Focusing on the reward circuitry and mu-opioid receptor. MedComm.

[88] Kurita G.P., Sjøgren P., Klepstad P., Mercadante S. (2019). Interventional Techniques for the Management of Cancer-Related Pain: Clinical and Critical Aspects. Cancers.

[89] Habib M.H., Schlögl M., Raza S., Chwistek M., Gulati A. (2023). Interventional pain management in cancer patients-a scoping review. Ann. Palliat. Med..

[90] Mao J.J., Ismaila N., Bao T., Barton D., Ben-Arye E., Garland E.L., Greenlee H., Leblanc T., Lee R.T., Lopez A.M. (2022). Integrative Medicine for Pain Management in Oncology: Society for Integrative Oncology-ASCO Guideline. J. Clin. Oncol..

[91] Swarm R.A., Youngwerth J.M., Agne J.L., Anitescu M., Are M., Buga S., Butler T., Chwistek M., Cleary J., Copenhaver D. (2025). Adult Cancer Pain, Version 2.2025, NCCN Clinical Practice Guidelines In Oncology. J. Natl. Compr. Cancer Netw..

[92] Calapai F., Mondello E., Mannucci C., Sorbara E.E., Gangemi S., Quattrone D., Calapai G., Cardia L. (2021). Pain Biomarkers in Cancer: An Overview. Curr. Pharm. Des..

[93] Mackey S., Aghaeepour N., Gaudilliere B., Kao M.C., Kaptan M., Lannon E., Pfyffer D., Weber K. (2025). Innovations in acute and chronic pain biomarkers: Enhancing diagnosis and personalized therapy. Reg. Anesth. Pain Med..

[94] Carlson C.L. (2016). Effectiveness of the World Health Organization cancer pain relief guidelines: An integrative review. J. Pain Res..

[95] Hui D., Bruera E. (2014). A personalized approach to assessing and managing pain in patients with cancer. J. Clin. Oncol..

[96] Raad M., López W.O.C., Sharafshah A., Assefi M., Lewandrowski K.U. (2023). Personalized Medicine in Cancer Pain Management. J. Pers. Med..

[97] Nijs J., Lahousse A., Fernández-De-Las-Peñas C., Madeleine P., Fontaine C., Nishigami T., Desmedt C., Vanhoeij M., Mostaqim K., Cuesta-Vargas A.I. (2023). Towards precision pain medicine for pain after cancer: The Cancer Pain Phenotyping Network multidisciplinary international guidelines for pain phenotyping using nociplastic pain criteria. Br. J. Anaesth..

[98] Giraud F., Pereira E., Anizon F., Moreau P. (2021). Recent Advances in Pain Management: Relevant Protein Kinases and Their Inhibitors. Molecules.

[99] Yousuf M.S., Shiers S.I., Sahn J.J., Price T.J. (2021). Pharmacological manipulation of translation as a therapeutic target for chronic pain. Pharmacol. Rev..

[100] Mantyh P.W., Koltzenburg M., Mendell L.M., Tive L., Shelton D.L. (2011). Antagonism of nerve growth factor-TrkA signaling and the relief of pain. Anesthesiology.

[101] Sopata M., Katz N., Carey W., Smith M.D., Keller D., Verburg K.M., West C.R., Wolfram G., Brown M.T. (2015). Efficacy and safety of tanezumab in the treatment of pain from bone metastases. Pain.

[102] Felix R., Corzo-Lopez A., Sandoval A. (2025). Voltage-Gated Ion Channels in Neuropathic Pain Signaling. Life.

[103] Coluzzi F., Scerpa M.S., Alessandri E., Romualdi P., Rocco M. (2025). Role of TRP Channels in Cancer-Induced Bone Pain. Int. J. Mol. Sci..

[104] Ghane G., Karimi R., Chekeni A.M., Darvishi M., Imani R., Vafaeinezhad F.Z. (2025). Pain Management in Cancer Patients With Artificial Intelligence: Narrative Review. Scientifica.

[105] Scholten S., Glombiewski J.A. (2025). Enhancing psychological assessment and treatment of chronic pain: A research agenda for personalized and process-based approaches. Curr. Opin. Psychol..

[106] Xu J., Liu X., Zhao J., Zhao J., Li H., Ye H., Ai S. (2025). Comprehensive Review on Personalized Pain Assessment and Multimodal Interventions for Postoperative Recovery Optimization. J. Pain Res..

[107] Adam R., de Bruin M., Burton C.D., Bond C.M., Giatsi Clausen M., Murchie P. (2018). What are the current challenges of managing cancer pain and could digital technologies help?. BMJ Support. Palliat. Care.

